# Pharmaceutical Industry in Syria

**Published:** 2010-08-25

**Authors:** D Kutaini

**Affiliations:** ‘Carol Davila’ University of Medicine and PharmacyRomania

**Keywords:** pharmaceutical industry, development

## Abstract

The aim of this article is to present the development of the pharmaceutical industry in Syria using national and international public data sources. At the end of the 80ies, the pharmaceutical industry in Syria was very poor, covering 6% of the national needs. In less than 20 years, with the government support in terms of legal frame and strategic political engagement, the Syrian pharmaceutical industry finally covered almost 90% of the national needs, in terms of drugs, and exported drugs in around 52 Arabian countries. Beyond covering the local market, the main added values of this huge development consisted in exporting drugs in amount of 150 million dollars per year and providing jobs for 17000 Syrian people, out of which around 85% are women. Strong and weak points of the pharmaceutical sector are taken into consideration in the article and further interventions to support a sustainable development are proposed by the author.

## Introduction

The state of health constitutes a key factor for productivity and the development of the population both socially and professionally, but also for the welfare and psychological comfort at an individual level. States worldwide, irrespective of their economical power, try to insure a better health service taking into consideration the direct relationship between health and productivity, but also the overall cost. 

In a different view, health services are an indispensable premise for insuring the state of health in the way defined by the World Health Organization – as a state of physical, psychical and social well–being.

Until the mid–80s, the simple basic condition required for manufacturing medicines were not available in Syria. Syria relied entirely on importing the medicines, having only two laboratories – Tamiko and Dimas – that belonged to the governmental sector. These two companies used only 6% of the requirements to cover the local market. Therefore, with the help and encouragement of the government, the private sector started to invest in the pharmaceutical industry by establishing of new plants and laboratories under the supervision and control of the Ministry of Health that cooperated with international organizations and pharmaceutical laboratories in implementing an intensive program meant to update all the aspects of the Pharmaceutical industry through the development of legislative and regulatory systems. Laboratories and production lines have been designed according to the conditions set by the World Health Organizations and the Global Companies. The regulations of good manufacturing practice (GMP) which were not known in Syria before 1988 became commonly used. A series of ISO standards for quality management systems (ISO 900), manufacturing of pharmaceutical products, storage conditions and environment management (ISO 1400) were implemented. 

## Achievements

The number of pharmaceutical factories in Syria has reached 63, producing 5700 types of products and offering jobs to about 17000 workers.

Today, we can say that the pharmaceutical industry has become one of the most important pillars of economic and social development in Syria. The local drugs production covers almost 90% of the national needs of Syria.

A plan meant to promote the production of generic drugs for cancer treatment, medicines related to blood diseases, vaccines, etc., and to cover 98 – 99% of the Syrian health sector needs is being implemented. According to this plan, the above–mentioned products will be manufactured locally within the next two years.

**The basic elements that make the pharmaceutical industry contribute to the national GDP are:**

1. Covering the local market and reducing the import of medicines, which reached a minimum of 700 million dollars.
2. Production reached almost 600 million dollars; the local market consumes more than 400 million dollars and the rest is sent for export.
3. Employing over 17000 workers of different qualification levels, university graduates, technicians and labors, out of which 85% are women.
4. Using modern and high technologies and implementing the principle of the high quality.
5. Running the pharmaceutical sector and related industry.

Due to the intensive ongoing training and rehabilitation programs, there are a large number of qualified workers with very high level of efficiency in the Pharmaceutical industry in Syria; over 25% of them are university graduates.

## Output of the Pharmaceutical Industries

Following the coefficient of the Syrian medicine on the map of the pharmacological treatment, we have found that with a few exceptions, most of the products meet the international standard systems. The exceptions are because either the products are new or they are protected by Patent Paw, and patent owning companies do not usually grant Production License to other companies, especially in the developing countries. Nevertheless, and as a result of the success achieved by the laboratories in developing new and modern techniques/technologies, lots of the major international pharmaceutical companies started to grant the manufacturing concession to a number of 16 Syrian laboratories. Over 58 international companies are now giving licenses for almost 390 products/formulas to be produced in Syria – a total of 8.5% of the whole products that are manufactured locally. Interaction and better communication between Syrian and foreign laboratories will allow access to a better exchange of information that will lead to an increasing rate of industrialization.

## Advanced position despite the challenges

Although Syria holds the second place in pharmaceutical production in the Arab world, exporting to more than 52 countries, there are still many challenges the pharmaceutical industry in Syria has to face:

1. Covering more than 90% of the pharmaceutical needs locally and nationally;
2. Availability of raw materials in the local market, achieving autonomy in the national industry;
3. Reviewing the medicines pricing base in accordance with the international specifications and within the moderate price range, elimination of foreign medicine, which are very expensive and helping producers to continue producing.
4. Lack of transparency and information about this type of business, the discrimination as far as the taxes and the absence of technical aspects to the certificate of origin are concerned.

Advantages are viability, competitiveness, fast development, ability to cover the local market, capability to reduce the import of medicines that had raised to 700 million dollars annually, capacity to produce drugs for more than 400 million dollars for the local market and 150 million dollars for export annually, possibility to offer about 17000 job opportunities. 

Disadvantages of this system might be the development and growth of the pharmaceutical industry is done more horizontally than vertically; lack of attention to the production of the raw materials, which are still imported from abroad; weak cooperation between Syrian pharmaceutical companies and weakness in the pricing mechanisms.

**Development initiatives and strategy through the following actions:**

- Establishment of a national observation office to collect information about the pharmaceutical and internationall markets in close cooperation with the Ministry of Foreign Affairs and the diplomatic missions abroad.
- Establishment of a national center of excellence for recognition and equivalence of diplomas/studies in collaboration with the Ministries of Education and Health.
- Solving problems related to the product price in the country of origin, which is an obstacle in obtaining favorable export prices of the products
- Encourage exporters through tax exemption to ensure competitive prices for products
- Issuance of a set of rules /decisions such as licenses for the organization of pharmaceutical plants according to the requirements, re–evaluation of custom duties and services in the countryside.
- Preparing to compete based on the specifications and quality rather than quantity.
- The necessity to apply and follow the regulations of good manufacturing practice system in all pharmaceutical laboratories that will make the Syrian products competitive not only in the domestic market but also internationally.
- There should be incentives such as loans and industrial land and legislative development systems, pricing, and good investment in the pharmaceutical sector with reference to a simple price advantage, therefore external agreements must be signed in order to unify the export price of the Syrian medicines to help it get a prominent place in the world.
- Establishment of a national independent department in charge of Pharmaceutical industries, affiliated to the Ministry of Health and having full power to make decisions that aim to the improvement of product quality, development of Syrian pharmaceutical industry to such high standards that the domestic product could cover all the consumers’ needs and be comparable in quality with the best products produced by global competitors.
